# Automated Head Tissue Modelling Based on Structural Magnetic Resonance Images for Electroencephalographic Source Reconstruction

**DOI:** 10.1007/s12021-020-09504-5

**Published:** 2021-01-27

**Authors:** Gaia Amaranta Taberna, Jessica Samogin, Dante Mantini

**Affiliations:** 1 Research Center for Motor Control and Neuroplasticity, KU Leuven, Tervuursevest 101, 3001 Leuven, Belgium; 2 Brain Imaging and Neural Dynamics Research Group, IRCCS San Camillo Hospital, Venice, Italy

**Keywords:** Head modelling, Magnetic resonance imaging, Head segmentation, Tissue probability mapping, Image processing, Source localization

## Abstract

In the last years, technological advancements for the analysis of electroencephalography (EEG) recordings have permitted to investigate neural activity and connectivity in the human brain with unprecedented precision and reliability. A crucial element for accurate EEG source reconstruction is the construction of a realistic head model, incorporating information on electrode positions and head tissue distribution. In this paper, we introduce MR-TIM, a toolbox for head tissue modelling from structural magnetic resonance (MR) images. The toolbox consists of three modules: 1) *image pre-processing* – the raw MR image is denoised and prepared for further analyses; 2) *tissue probability mapping* – template tissue probability maps (TPMs) in individual space are generated from the MR image; 3) *tissue segmentation* – information from all the TPMs is integrated such that each voxel in the MR image is assigned to a specific tissue. MR-TIM generates highly realistic 3D masks, five of which are associated with brain structures (brain and cerebellar grey matter, brain and cerebellar white matter, and brainstem) and the remaining seven with other head tissues (cerebrospinal fluid, spongy and compact bones, eyes, muscle, fat and skin). Our validation, conducted on MR images collected in healthy volunteers and patients as well as an MR template image from an open-source repository, demonstrates that MR-TIM is more accurate than alternative approaches for whole-head tissue segmentation. We hope that MR-TIM, by yielding an increased precision in head modelling, will contribute to a more widespread use of EEG as a brain imaging technique.

**Supplementary Information:**

The online version contains supplementary material available at 10.1007/s12021-020-09504-5.

## Introduction

Electroencephalography (EEG) measures the variation of electrical potentials over the scalp, which are generated by neural activity. Due to its high temporal resolution, this technique is particularly suited for the investigation of neural dynamics during task performance as well as during resting state (Mantini et al. 2007; Michel and Murray 2012; Liu et al. 2017). Most EEG studies conduct analyses of neural dynamics in the sensor space. However, recent technological developments have also enabled the use of EEG signals for the reconstruction of neural sources. When source localization is performed, EEG can be used as a non-invasive neuroimaging technique, in alternative to magnetoencephalography (Hipp et al. 2012; Michel and Murray 2012) and functional magnetic resonance imaging (Ganzetti and Mantini 2013; Mantini et al. 2007; Marino et al. 2019; Samogin et al. 2019). A first crucial element to ensure the reliability of EEG source localizations is the use of high-density electrode montages, including more than hundred sensors (Liu et al. 2017; Samogin et al. 2019; Seeber et al. 2019). It is also fundamental that an accurate individual head model is built, integrating information on the electrode positions over the scalp and on the spatial distribution of head tissues (Michel et al. 2004). This model is used to estimate how neural activity propagates from the sources inside the brain to the sensors, providing crucial information for an accurate source localization (Hallez et al. 2007).

Increasingly accurate techniques for head modelling have become available in the course of the years. Initially, the human head was modelled as a simple homogeneous sphere, and subsequently as three (or four) concentric spherical layers distinguishing brain, skull and scalp (and cerebrospinal fluid – CSF) compartments (Berg and Scherg 1994; Homma et al. 1994; Sun 1997). These approaches required to make strong approximations concerning the positioning of the EEG electrodes over the scalp surface. The extent of these approximations reduced considerably when magnetic resonance (MR) images started being used to estimate scalp, skull and brain meshes for individual participants. Boundary element methods (BEMs) that relied on those meshes for the construction of the head model were developed (Akalin-Acar and Gencer 2004; Hamalainen and Sarvas 1989). An important limitation associated with the use of BEMs is that the meshes cannot intersect each other. This leads to an oversimplification of skull and brain layers, as well as the impossibility to separate grey and white matter (GM/WM) and to appropriately model the cerebrospinal fluid (CSF).

From this standpoint, an important advancement in head modelling for EEG analysis was brought by the introduction of finite element methods (FEMs) (Lew et al. 2009; Rullmann et al. 2009; Wolters et al. 2007) and finite difference methods (FDMs) (Cuartas Morales et al. 2019; Hallez et al. 2005; Saleheen and Ng 1997). Both these approaches use the structural MR image to define flexible arrays of hexahedral or tetrahedral elements, based on which the propagation of neural currents in the head can be computed. It should be considered, however, that each hexahedral/tetrahedral element needs to be associated with a conductivity value that is characteristic of the specific tissue it belongs to (Akhtari et al. 2002; Baumann et al. 1997; Haueisen et al. 1997). To address this requirement, the structural MR image needs to be accurately segmented into the different head tissues. There is no intrinsic limitation on the number of head tissues that can be defined, provided that a specific conductivity value can be assigned to each tissue.

The introduction of FEMs and FDMs has given strong impulse to the use of highly realistic head models, requiring the segmentation of a structural MR image to accurately define the head tissue distribution (Montes-Restrepo et al. 2014; Ramon et al. 2006; Vatta et al. 2009). Over the years, MR segmentation methods have been largely improved, and extended to higher numbers of tissue classes. Wagner et al. (2014) proposed a semi-automated six-tissue (GM, WM, CSF, compact bone, spongy bone and skin) segmentation method, which was based on multimodal MR imaging data. Holdefer et al. (2006) defined eight tissue compartments (GM, WM, CSF, compact bone, spongy bone, blood, soft tissues and skin), but their approach yet required heavy manual intervention. Li et al. (2016) presented an automated segmentation method with seven tissues (GM, WM, CSF, eyes, bone, flesh and air), based on both MR and computed tomography (CT) images. It should be considered, however, that the availability of both MR and CT images of the same participant is very uncommon in EEG studies.

An alternative approach to the segmentation of MR images in individual space is the use of already segmented template MR images. We previously used this approach to build a head model including twelve tissue classes (brain GM, cerebellar GM, brain WM, cerebellar WM, brainstem, CSF, spongy bone, compact bone, muscle, fat, eyes and skin) (Liu et al. 2017; Liu et al. 2018; Samogin et al. 2019; Zhao et al. 2019). An advantage of this solution is that a high number of tissue classes can be easily obtained (Iacono et al. 2015); however, spatial distortions are likely to occur, considering that the segmented template image needs to be warped to individual space.

Our recent studies based on high-density EEG showed that source localizations can be strongly improved by using very realistic head models (Liu et al. 2017; Liu et al. 2018). In light of those findings, we aimed at further improving the current methods for head tissue modelling based on structural MR images. In this study, we introduce MR-TIM, which stands for *MR-based head tissue modelling*. This is a software toolbox to perform an automated segmentation of a T1-weighted MR image in 12 tissue classes. The performance of MR-TIM was evaluated both qualitatively and quantitatively. In particular, we used MR images from individual participants as well as segmented images associated with an MR template. In this manner, we were able to provide evidence for an improvement in segmentation precision as compared to alternative solutions.

## Methods

MR-TIM was written in the MATLAB environment (MathWorks, Natick, MA, US), and subsequently integrated in the SPM12 software package (http://www.fil.ion.ucl.ac.uk/spm/software/spm12) to be used as a toolbox. Its code, which is available under a GNU General Public License, can be downloaded from NITRC at https://www.nitrc.org/projects/mr-tim, and from GitHub at https://github.com/gtaberna/mrtim.

### Toolbox Overview

**Fig. 1:**
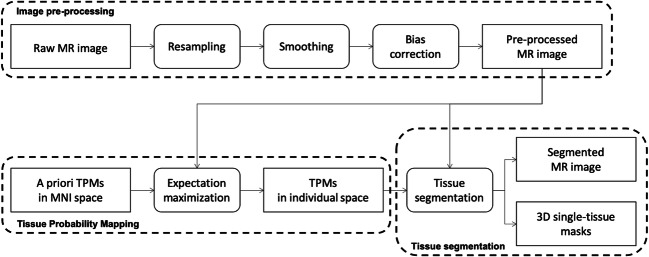
Workflow of the MR-TIM analysis for structural MR images. The analysis is accomplished through three main modules: image pre-processing, tissue probability mapping and tissue segmentation. The final output is a segmented image and (optionally) a 3D mask for each segmented tissue

MR-TIM is divided into three main modules: 1) image pre-processing; 2) tissue probability mapping; 3) tissue segmentation (Supplementary Fig. 1). With the first module, the MR image is denoised and prepared for further analyses; with the second module, tissue probability maps in individual space are generated from the MR image; with the third module, information from all the tissue probability maps is integrated such that each voxel in the MR image is assigned to a specific tissue (Fig. 1). Settings and parameters for each module can be selected through a graphical user interface integrated in SPM12 (Supplementary Fig. 1). A detailed description of the modules is provided in the following sections.

#### Image Pre-Processing

The raw T1-weighted MR image, in NIFTI format and with the name *anatomy.nii*, is pre-processed to reduce background noise and to correct for intensity inhomogeneity. First of all, the image is resampled at 1 mm isotropic resolution, and spatially smoothed. Next, intensity inhomogeneity is attenuated using the bias correction tool in the SPM12 segmentation toolbox. Finally, all the intensities below a selected intensity threshold are set to zero. The spatial smoothing kernel and the intensity threshold are set to 1 mm full width half maximum and 5% of the maximum image value, respectively. The user can tune these parameters through the graphical user interface. The pre-processed MR image is saved as *anatomy_prepro.nii*.

#### Tissue Probability Mapping

The pre-processed MR image is then given as input to the segmentation tool of SPM12, along with the tissue probability maps (TPMs) in the Montreal Neurological Institute (MNI) standard space. The TPM is a 3D image with intensity values ranging between 0 and 1, representing the likelihood that each pixel belongs to the specific tissue class. The TPMs in MNI space implemented in MR-TIM are derived from the MIDA model (Iacono et al. 2015) and are associated with the following twelve tissue classes: brain GM, cerebellar GM, brain WM, cerebellar WM, brainstem, CSF, spongy bone, compact bone, eyes, muscle, fat, and background (Liu et al. 2017; Liu et al. 2018) (see Supplementary Fig. 3). The output of the SPM12 segmentation tool is a set of TPMs in individual MR space. These TPMs, each corresponding to a TPM in MNI space, are estimated based on the intensity profile in the pre-processed MR image. They are saved in a 4D image called *anatomy_prepro_tpm.nii*.

#### Tissue Segmentation

The TPMs in individual space are used as priors for the definition of the tissue classes. Morphological and intensity-related operations are used to generate a set of 3D masks, one for each tissue. These masks are generated in a sequential manner (from the brain to the skull, to the soft head tissues), and finally integrated in a single 3D image containing the segmented head. A priori information on the anatomy of the human head is used to constrain the tissue spatial distribution. Specifically, the CSF is set to fill the ventricles and the subarachnoid space around the brain (GM and WM); the compact bone is set to surround the external surface of the CSF and the whole surface of the spongy bone. Also, the soft head tissues (muscle and fat) and the skin lay outside the skull and brain. Once all 3D masks, one for each tissue class, are created, they are integrated in the same space. A maximum likelihood approach based on the TPMs is used to resolve cases of overlap between tissues, or to fill gaps. The final output of the tissue segmentation is a 3D image named *anatomy_prepro_segment.nii*, with voxel values in the range from 0 to 12. The value 0 defines the background area external to the head and the other labels define the twelve tissues. Optionally, the user can choose to save, along with this labelled image, also the binary masks of each segmented tissue. If needed, 3D meshes can be generated using external software that can read and process NIFTI files.

### Validation

#### MR Datasets

We used five structural MR datasets for the validation of our method. The first dataset contained 6 MR images collected in healthy young volunteers, on which we primarily conducted qualitative analyses. Then we moved to template MR data from an open-source online database, which also contained a segmented image. This permitted us to conduct qualitative as well as quantitative analyses. Finally, we performed a large-scale validation on MR images from three other online databases, which were collected using different MR scanners, in healthy participants and patients of different age.

##### Individual MR Data

Each participant gave written informed consent to the experimental procedures, which were approved by the Ethics Committee of the KU Leuven. For each participant, a structural T1-weighted MR image was collected with a Philips Achieva 3.0 T MRI system and a 32-channel head coil. The images were acquired with a magnetization-prepared rapid-acquisition gradient-echo (MPRAGE) sequence with the following parameters: TR = 9.6 ms; TE  =  4.6 ms; voxel size  =  1  ×  1  ×  1.2 mm^3^; field of view  =  250  ×  250; 160 coronal slices.

##### Template MR Data

We also used data from the Scientific Computing and Imaging Institute (SCI) Head Model of the University of Utah (https://www.sci.utah.edu/sci-headmodel.html) (Warner et al. 2019). This open-source repository contains a high-resolution (1 mm isotropic) T1-weighted image, as well as a seven-layer whole-head segmented image including the following tissue classes: GM, WM, CSF, skull, sinus, eyes, and scalp. This segmented image was obtained following a manual procedure using the FSL toolbox (Jenkinson et al. 2012) and the Seg3D software (NIH Center for Integrative Biomedical Computing, University of Utah, www.seg3d.org).

##### Database MR Data

Finally, we used MR images from three databases: the IXI database of the Imperial College of London (https://brain-development.org/ixi-dataset/); the Autism Brain Imaging Data Exchange (ABIDE) database (http://fcon_1000.projects.nitrc.org); the SchizConnect database (http://schizconnect.org). The IXI database contains structural T1-weighted images collected in participants with age ranging between 20 and 86 years, using either a Philips Intera 3.0 T or a Philips Gyroscan Intera 1.5 T MRI system. For our validation, we extracted a total of 40 MR images: (a) 10 from young participants (age 20–35 years), acquired with the 3.0 T scanner; (b) 10 from young participants (age 20–35 years), acquired with the 1.5 T scanner; (c) 10 from elderly participants (age 60–75 years), acquired with the 3.0 T scanner; (d) 10 from elderly participants (age 60–75 years), acquired with the 1.5 T scanner. The ABIDE database contains structural T1-weighted images collected in patients with autism spectrum disorder (ASD); we specifically extracted MR images from 10 participants (age 18–25), collected using a Philips Achieva 3.0 T MRI system. The SchizConnect database contains structural T1-weighted images collected in patients with schizophrenia; we specifically extracted MR images from 10 participants (age 19–66), collected using a Siemens Trio Tim 3.0 T MRI system.

#### Method Assessment

We initially conducted a qualitative assessment of MR-TIM, applying the method to the MR data from individual participants. Three other methods were used for comparison. The first one, which was earlier proposed by our group, segments the MR image by warping a precomputed 12-layer segmentation in MNI space to individual space (Liu et al. 2017). In the following, we will refer to this approach as ‘*warping of template segmentation’*, or WTS. The second alternative method is based on the segmentation scripts available in the Fieldtrip toolbox (http://www.fieldtriptoolbox.org) (Oostenveld et al. 2011), which is used by a wide community of researchers. The FieldTrip segmentation can yield up to five tissue classes (GM, WM, CSF, skull and scalp), but in standard settings it produces the three tissue classes to be used for BEMs (brain, skull, scalp). The third method is integrated in the BrainStorm software (http://neuroimage.usc.edu/brainstorm) (Tadel et al. 2011), and relies on tissue segmentation by FreeSurfer (https://surfer.nmr.mgh.harvard.edu) (Fischl 2012). The output of the segmentation produced by BrainStorm has similar format as the one of FieldTrip. For qualitative comparisons of the results produced by MR-TIM, WTS, FieldTrip and BrainStorm, meshes were created using 3DSlicer (https://www.slicer.org) (Kikinis et al. 2014).

It should be noted that no ground truth is available for individual MR images. To address this limitation, we also analysed data available from the SCI Head Model, which included both a T1-weighted MR image and its corresponding segmentation. First, we conducted a qualitative analysis of the SCI MR data, processed using MR-TIM, WTS, FieldTrip and BrainStorm, respectively. Then, we used the segmented image from the SCI Head Model as reference, and we performed quantitative analyses. Considering that the SCI segmentation contained seven tissue classes, the output of MR-TIM and WTS was post-processed, combining brain GM with cerebellar GM, brain WM with cerebellar WM and brainstem, spongy bone with compact bone (skull), muscle with fat and skin (scalp). Since the segmentation produced by FieldTrip and BrainStorm contained maximum five classes, these two methods were not included in the quantitative analysis. For each tissue class in the SCI Head Model (excluding the sinus) and for the background (BG), we defined three indices to quantify the precision of MR-TIM and WTS. The first index is the Spatial Overlap index *K* defined as $K=\frac{\left|A\cap B\right|}{\left|B\right|}$ (Cardenes et al. 2009); the second one is the Dice index *D*, defined as $D=\frac{2\ \left|A\cap B\right|}{\left|A\right|+\left|B\right|}$ (Dice 1945); the third one is the Jaccard index *J*, defined as $J=\frac{\left|A\cap B\right|}{\left|A\cup B\right|}$ (Jaccard 1912). In the formulas above, *A* is the test image and *B* is the reference image, whereas *A* ∪ *B* and *A* ∩ *B* are the union and intersection of *A* and *B*, respectively. The notation |∙| indicates the sum of voxels included in the image. All the three indices defined above are in the range between 0 and 1, with 1 reflecting perfect overlap between images and 0 no overlap.

Finally, we processed the MR images from the IXI, ABIDE and SchizConnect databases with both MR-TIM and WTS, so to obtain 12-layer segmentations. They were scored by two independent raters, who did not have any information concerning how the specific MR image was collected and processed, but were not blinded to which tissue should have been assessed. For each tissue class and each segmentation method, the raters assigned a qualitative segmentation score, defined as follows: *excellent*, *good*, *doubtful* or *failed* (Klapwijk et al. 2019). First, we quantified the inter-rater reliability of the segmentation scores for IXI, ABIDE and SchizConnect databases, respectively, using the linear weighted Cohen’s kappa coefficient κ (Cohen 1960). In addition, the segmentation scores obtained from the IXI database were analyzed using a linear regression model with three factors: age (young vs. elderly), scanner type (3 T scanner vs. 1.5 T scanner) and segmentation method (MR-TIM vs. WTS). In this manner, we tested if and to what extent each of the factors contributed to the differences across segmentation scores.

## Results

**Fig. 2:**
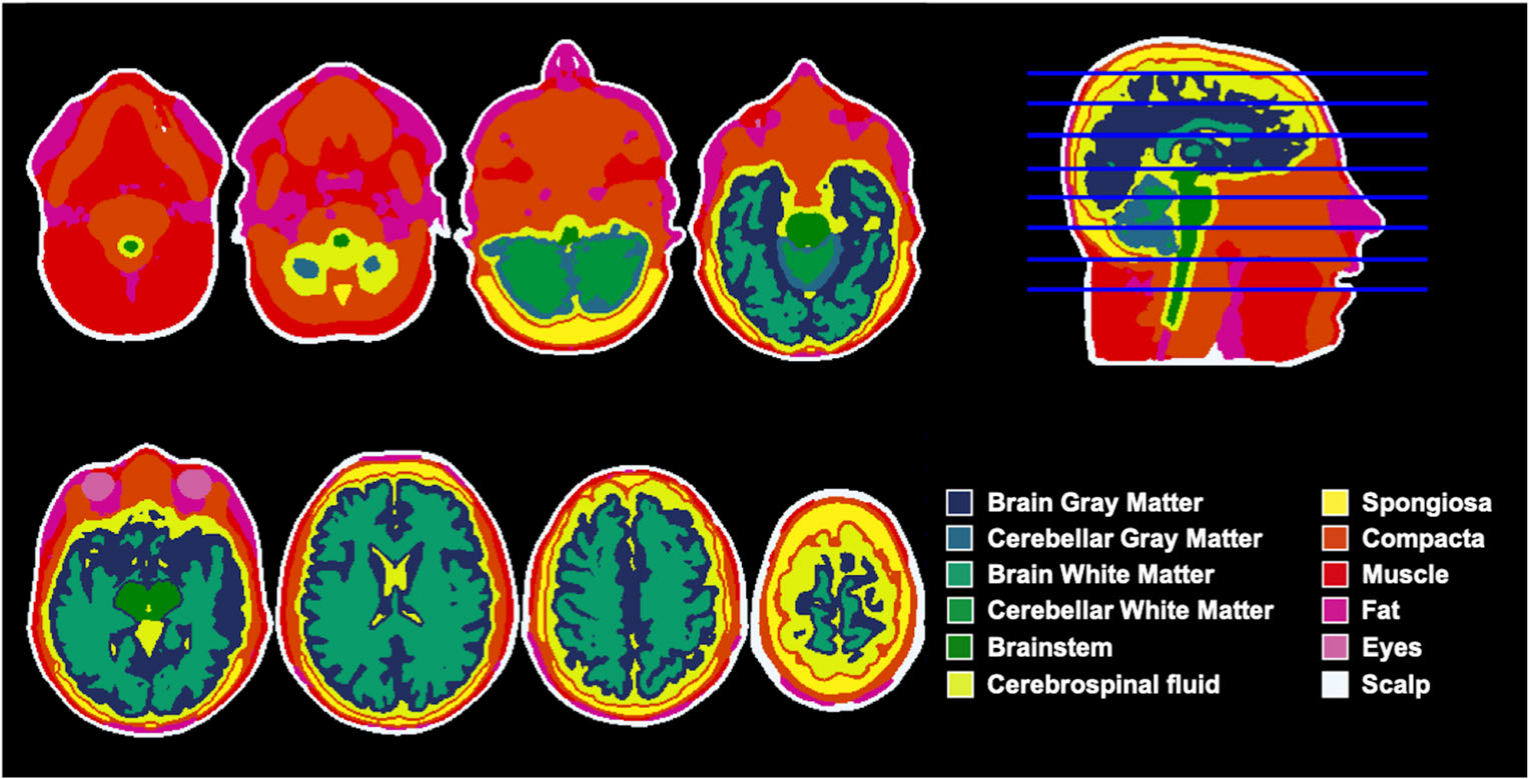
Sample output of whole-head automated segmentation produced by MR-TIM. The results obtained on the MR image from subject S01 are shown in volumetric space. The 12 tissue classes are represented with different colours

**Fig. 3:**
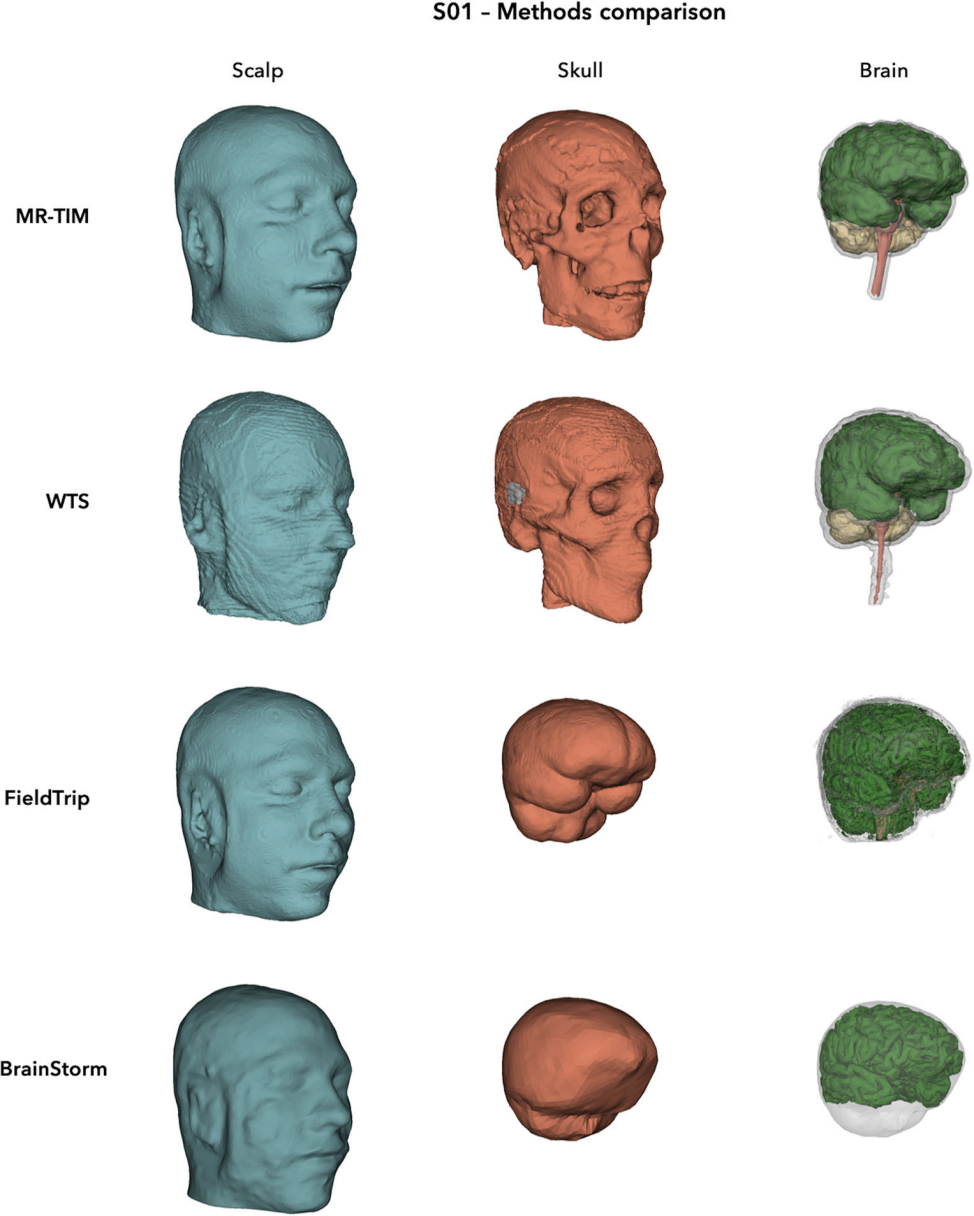
3D surfaces of scalp, skull and brain, estimated by MR-TIM, WTS, FieldTrip and BrainStorm using an individual MR image. The results were obtained using the MR image from subject S01

Using MR-TIM, we performed an automated whole-head segmentation in twelve tissue classes from individual MR images (see Fig. 2 and Supplementary Fig. 2 for a detailed example). A valid segmentation was obtained for all six MR images included in the study. Aiming at comparing the output produced by MR-TIM with other methods, we processed the individual MR images also with WTS, FieldTrip and BrainStorm. We built and plotted 3D surfaces for the five tissues generated by all four methods: scalp, skull, GM, WM and CSF. Visual inspection of the results evidenced a relative good correspondence between the skin layers generated by MR-TIM, FieldTrip and BrainStorm, and between skull, GM and WM layers generated by MR-TIM and WTS (Fig. 3 and Supplementary Figs. 4–7). Conversely, relatively large spatial distortions were observed in the skin layer produced by WTS. Also, the skull layer generated by FieldTrip and BrainStorm appeared to be very smooth and with oversimplified geometry.

**Fig. 4:**
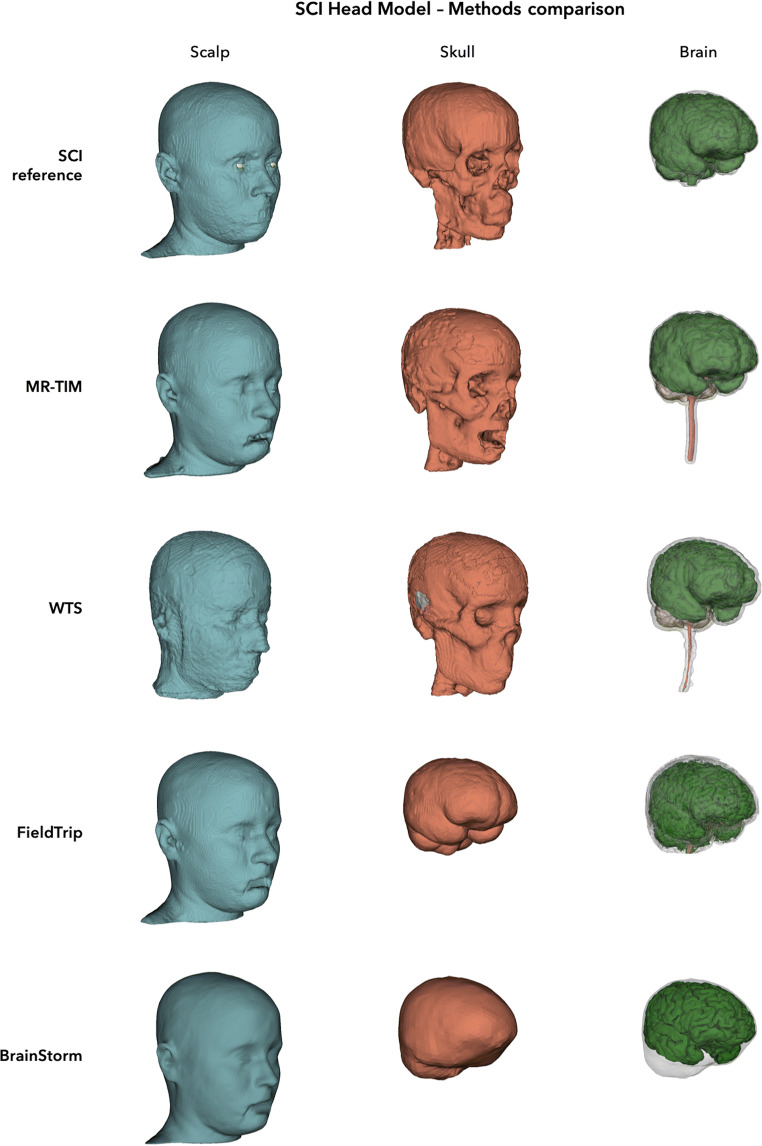
3D surfaces of scalp, skull and brain estimated by MR-TIM, WTS, FieldTrip and BrainStorm using a template MR image. The results were obtained using the MR image from the SCI Head Model

**Fig. 5:**
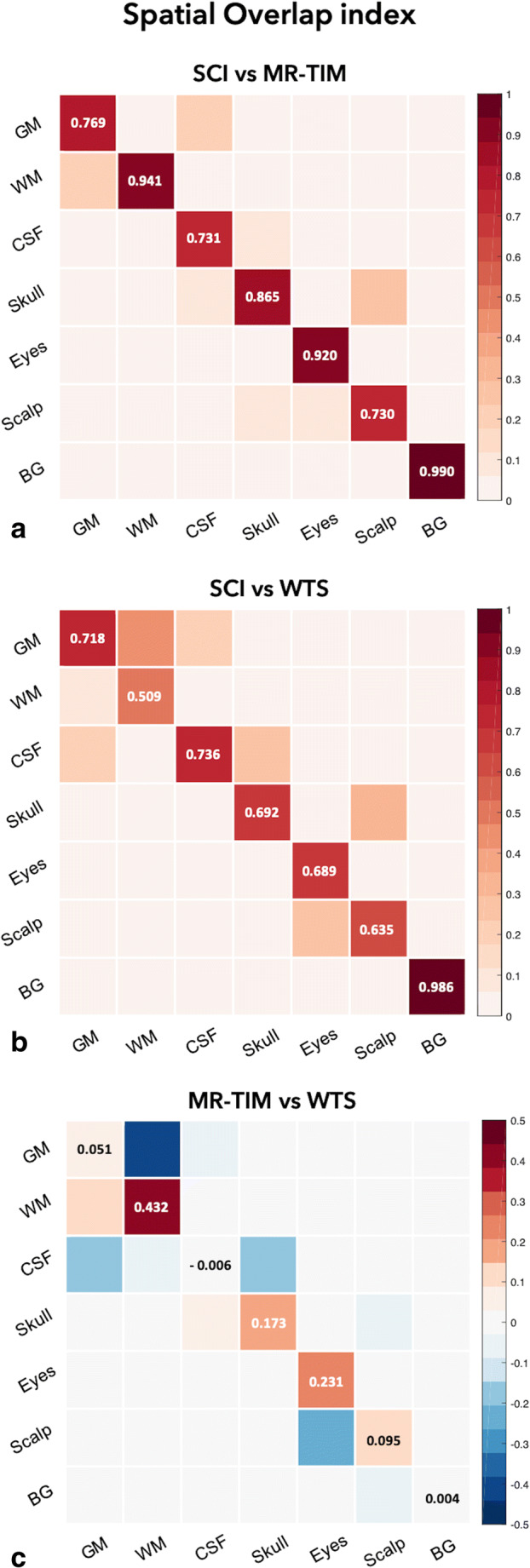
Tissue estimation performance of MR-TIM and WTS, measured using the Spatial Overlap index. The SCI Head Model segmentation is used as reference, and compared against the results produced by (A) MR-TIM and (B) WTS. Each value in the confusion tables represents the Spatial Overlap index between tissue masks. (C) The difference in the Spatial Overlap index obtained using MR-TIM and WTS, respectively, is shown as well. GM: grey matter; WM: white matter; CSF: cerebrospinal fluid; BG: background

Results in line with those obtained from individual MR images were obtained when MR-TIM, WTS, FieldTrip and BrainStorm were applied to the MR image of the SCI Head Model (Fig. 4). In this case, however, we could use the corresponding segmented image as reference, and could also conduct quantitative analyses for MR-TIM and WTS (Fig. 5). Specifically, we obtained an average Spatial Overlap index equal to 0.71 (range 0.51–0.99) for WTS and 0.86 (range 0.73–0.99) for MR-TIM (Fig. 5a-b). We found an improvement in the segmentation of MR-TIM compared to WTS for all tissue classes except for the CSF (Fig. 5c); more specifically, the major differences in terms of Spatial Overlap were observed for WM (+ 39.3%), eyes (+23.9%), scalp (+16.7%) and GM (+16.4%). The results that we obtained when assessing the segmentation performance with the Spatial Overlap index were corroborated by those produced using the Dice and Jaccard indices (Supplementary Figs. 8 and 9).

**Fig. 6:**
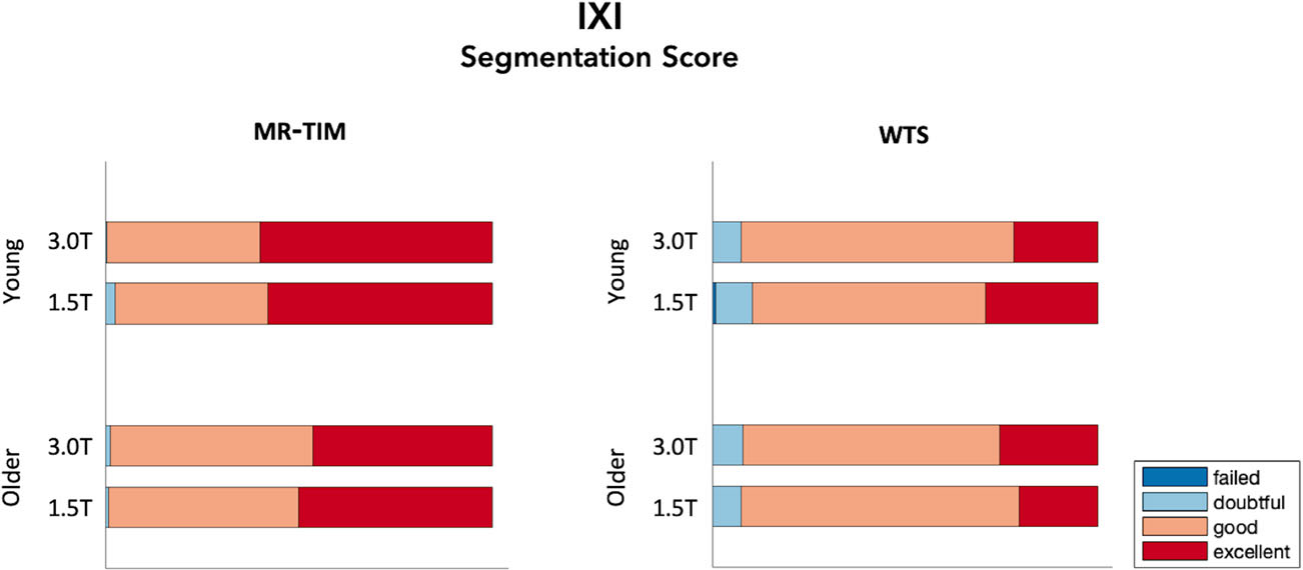
Qualitative segmentation scores based on the scale: *excellent*, *good*, *doubtful* and *failed*. Scores related to the 40 MR images from the IXI dataset. The stacked bar plots compare the scores for MR-TIM (left) and WTS (right) segmentation methods, divided per groups of: age of the participant, young (20–35 years old) and older individuals (60–75 years old); MR scanner type, 3.0 T and 1.5 T

The validation performed using the IXI database allowed us to examine the generalizability of the results from data collected with different scanners and in individuals with different age (Fig. 6). We observed overall high segmentation scores, with 42.2% and 35.5% *excellent*, for young and old participants respectively, as well as 52.6% and 60.1% *good*, respectively; 38.3% *excellent* and 57.4% *good* for the 3.0 T MR scanner, 39.4% *excellent* and 55.3% *good* for the 1.5 T MR scanner; 53.5% *excellent* and 45.2% *good* for MR-TIM, 24.2% *excellent* and 67.5% *good* for WTS. Linear regression analysis revealed significant differences between segmentation methods (*p* < 0.0001), with MR-TIM outperforming WTS. No significant differences were found between age groups (*p* = 0.128) and scanner types (*p* = 0.955). The analysis of individual tissue classes evidenced that the improvement of MR-TIM compared to WTS was to be primarily ascribed to the segmentation of grey matter, brainstem, skin and muscles. On the other hand, relatively less accurate segmentation for MR-TIM were observed for spongy bone, fat and eyes. MR-TIM yielded an improved segmentation of the CSF on the MR images from the IXI database, which is not in line with the results obtained from the SCI Head Model. Specifically, we obtained a rate of *excellent* evaluations for CSF across participants equal to 55.0% and 16.3% for MR-TIM and WTS, respectively. The segmentation scores of the two raters were in moderate/substantial agreement, with κ = 0.57 (95% C.I., 0.48–0.66).

**Fig. 7:**
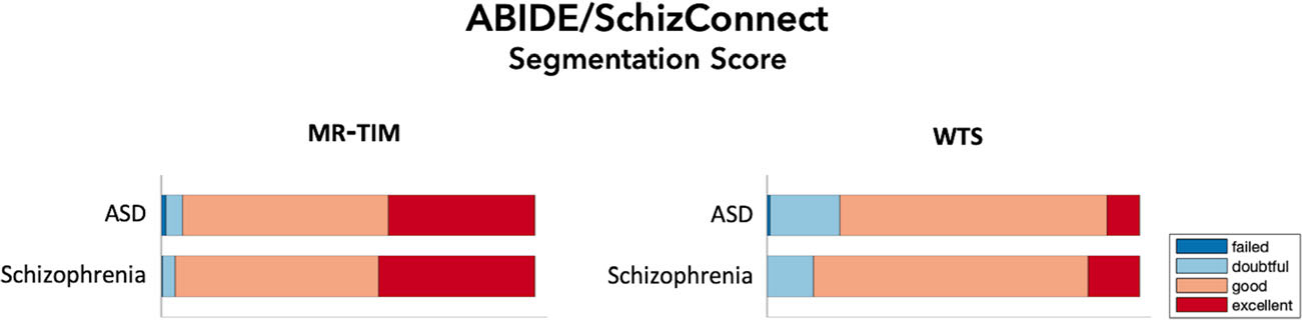
Qualitative segmentation scores based on the scale: *excellent*, *good*, *doubtful* and *failed*. Scores related to the 20 MR images from the ABIDE and SchizConnect databases. The stacked bar plots compare the scores for MR-TIM (left) and WTS (right) segmentation methods, divided per groups of mental disorder of the participant: autism spectrum disorders (ASD) and schizophrenia

When we segmented MR images of patients, we obtained comparable performance as compared to that of healthy individuals. We had overall high segmentation scores, with 24.0% *excellent* and 63.3% *good* for ASD patients, as well as 27.7% *excellent* and 64.2% *good* for schizophrenia patients. Also, we obtained 40.4% *excellent* and 54.8% *good* for MR-TIM, 11.3% *excellent* and 72.7% *good* for WTS (Fig. 7). Again, the segmentation scores of the two raters were in a moderate/substantial agreement, with κ = 0.52 (95% C.I., 0.34–0.70) for ABIDE and κ = 0.49 (95% C.I., 0.29–0.69) for SchizConnect.

## Discussion

Our previous work showed that an increased precision in head modelling can be crucial to ensure the accuracy of EEG source localizations (Liu et al. 2017; Liu et al. 2018), fostering the use of EEG as brain imaging technique (Samogin et al. 2019; Zhao et al. 2019). Aiming at providing a valid and reliable approach to head modelling, we previously proposed to register a 12-layer template segmentation to individual space (Liu et al. 2017), according to a WTS approach. Other existing solutions permit to obtain segmentations with a lower number of head tissues, and typically require manual fine-tuning (Holdefer et al. 2006; Li et al. 2016; Wagner et al. 2014). Although the WTS solution has its own merits, an important limitation needs to be considered: registration errors from template to individual space can inevitably occur, and primarily affect head tissue definition. Aiming at addressing this limitation, we developed MR-TIM, a toolbox that permits highly realistic segmentation of a structural MR image in 12 head tissue classes. Our validation confirmed that MR-TIM can reliably define head tissue classes in individual space. This permits to take full advantage of recent developments in forward modelling (Cuartas Morales et al. 2019), with positive impact on EEG source localizations (Michel et al. 2004).

### Analysis of Method Features and Performance

A large number of EEG source localization studies rely on BEMs for the creation of the leadfield matrix, which mathematically describes how neural currents in the cortex are related to the potentials measured by EEG sensors over the scalp (Michel et al. 2004). The use of BEMs typically requires the segmentation of head tissues in only three classes: brain, skull and scalp. FEMs and FDMs have however demonstrated a superior precision compared to BEMs for the calculation of the leadfield matrix, in particular when highly realistic head segmentations are used (Cuartas Morales et al. 2019; Hallez et al. 2007).

Manual or semi-manual approaches can be used to obtain highly realistic head segmentations (Briend et al. 2020; Holdefer et al. 2006; Wagner et al. 2014). However, these approaches can be very time-consuming, and are in particular operator-dependent. They are therefore unsuitable for use in large-scale analyses of EEG data, and do not ensure reproducibility of EEG imaging results even in studies with a relatively small number of participants. Alternatively, high-resolution segmented images of the human head have been proposed as an important aid for EEG forward modelling (Huang et al. 2016; Warner et al. 2019). Those images can be very helpful to conduct simulations or can be used in studies in which information on electrode positions is unavailable. It is evident, however, that a high precision in the calculation of the leadfield matrix can be achieved only when electrode positions are collected and are spatially aligned with the MR image of the same participant. It is for this reason that, in our previous studies, we have used the WTS approach. In particular, we warped a precomputed 12-layer segmentation in MNI space to individual space (Liu et al. 2017). We demonstrated that this WTS approach, in combination with FEM for the leadfield matrix calculation, can lead to a remarkable precision in the reconstruction of EEG brain networks (Liu et al. 2018).

The development of MR-TIM takes its origin from the observation that our solution for head tissue segmentation could be improved, possibly leading to an increased precision in EEG source reconstruction. Specifically, we aimed to develop a novel, completely automated technique that could segment head tissues in individual MR space. This solution, which we called MR-TIM, has three main steps (Fig. 1): the first one is aimed at standardizing the input MR image; the second one generates 12 distinct TPMs by combining topological and intensity information contained in the MR image; the third step integrates the information contained in the TPMs to assign each voxel in the MR image to one of the 12 tissue classes.

The quality of the segmentation produced by MR-TIM was satisfactory (Figs. 2-4), as to enable head modelling. In particular, the MR-TIM segmentation of the template MR image from the SCI Head Model showed a high degree of correspondence with the SCI segmented image (Figs. 4-5). With some exceptions, we observed good match between the single-tissue masks obtained using MR-TIM and WTS. Conversely, the tissue classes identified by FieldTrip (Oostenveld et al. 2011) and BrainStorm (Tadel et al. 2011) differed substantially from those of MR-TIM and WTS. In particular, the skull compartment was very smooth and did not seem to have realistic morphology (Figs. 3-4).

Upon close inspection, we found that MR-TIM presented discontinuities for some tissues, and particularly the skull. The reconstruction of the skull was probably the most challenging task, as the compact and spongy bone layers are very thin (Fig. 3-4). Quantitative analyses showed that the skull reconstruction of MR-TIM was more accurate than for WTS (Fig. 5).

We also found that WTS was characterized by spatial distortions for external head layers, including the skin (Fig. 3-4). This may be due to the fact that spatial normalization algorithms are optimized for the registration of the brain (Ashburner and Friston 1999; Friston et al. 1995), rather than of the whole head. Notably, we found differences between MR-TIM and WTS also for WM, with the first method showing better correspondence with the reference segmentation (Fig. 5). Also in this case, the result may be explained by an intrinsic difficulty in the spatial registration of subcortical regions.

The results obtained using MR images from the IXI database revealed that MR-TIM was not significantly influenced by the age of the participants and by the MR scanner system used for acquisition (Fig. 6). On the other hand, its use resulted in a significant improvement in segmentation performance as compared to WTS. Also, MR-TIM produced satisfactory results with MR images collected in patients with ASD and schizophrenia (Fig. 7), which are neurological conditions characterized by increased brain size and ventricular enlargement, respectively.

### Limitations and Future Work

A number of limitations of the study should be mentioned. First of all, the performance of MR-TIM was compared with only three other head segmentation methods based on T1-weighted MR images, WTS, FieldTrip and BrainStorm. Notably, MR-TIM has been developed for use with T1-weighted MR data, but it could be extended in the future to process also T2-weighted MR and CT data. This further development would then provide the opportunity to compare MR-TIM with other methods that rely also on T2-weighted MR (Wagner et al. 2014) or CT images (Li et al. 2016).

As a second limitation, we would like to mention that MR-TIM, in its current implementation, is not suited to process images collected in participants with brain lesions, as those produced by stroke, brain tumour or traumatic brain injury. The presence of a lesion in the brain, in particular if cortical, may have a strong impact on EEG source localizations (Irimia et al. 2013). For this reason, our future methodological work will be directed towards an upgrade of MR-TIM, such that the toolbox can be used with MR images from bran-lesioned patients.

A third limitation is the fact that reference segmentations were not available for the MR images from the IXI, ABIDE and SchizConnect databases. Accordingly, we could only conduct qualitative assessments of the MR-TIM output. We specifically used a four-step scale ranging from *failed* to *excellent*, to facilitate the comparability of our results with those of other studies (Klapwijk et al. 2019).

Finally, we would like to point out the fact that, once the segmentation has been performed, a specific conductivity value needs to be associated with each tissue for the creation of the head model (Michel et al. 2004). A large number of EEG studies rely on conductivity values from the literature (McCann et al. 2019). Nonetheless, a direct estimate of the conductivity values in individual participants would be highly desirable to increase the accuracy of EEG forward modelling, hence of EEG source localizations.

## Conclusion

In this study we have introduced MR-TIM, a toolbox to perform fully automated whole-head tissue segmentation from structural MR images. MR-TIM generates highly realistic 3D masks, five of which are associated with brain structures (brain GM, cerebellar GM, brain WM, cerebellar WM and brainstem) and seven with other head tissues (CSF, spongy bone, compact bone, eyes, muscle, fat and skin). Our validation, conducted on MR images collected in healthy volunteers and patients, as well as on an MR template image from an open-source repository, demonstrates that MR-TIM is more accurate than alternative approaches for whole-head tissue segmentation. We hope that MR-TIM, by yielding an increased precision in head modelling, will contribute to a more widespread use of EEG as a brain imaging technique.

## Information Sharing Statement

MR-TIM software is distributed according to a GNU General Public License, and is available for download at https://www.nitrc.org/projects/mr-tim and at https://github.com/gtaberna/mrtim.

## Supplementary Information

ESM 1 (DOCX 9138 kb)
