# Supplementary material for: Automated Head Tissue Modelling Based on Structural Magnetic Resonance Images for Electroencephalographic Source Reconstruction
Source: Neuroinformatics. 2021 Jan 27;19(4):585–96. doi: 10.1007/s12021-020-09504-5 (PMC8566646; doi:10.1007/s12021-020-09504-5)
Supplement: Supplementary file 1 — (DOCX 9138 kb) [file 12021_2020_9504_MOESM1_ESM.docx]

**Supplementary Material**

**Automated head tissue modelling based on structural magnetic resonance images for electroencephalographic source reconstruction**

Gaia Amaranta Taberna^1^, Jessica Samogin^1^, Dante Mantini^1,2^

^1^ Research Center for Motor Control and Neuroplasticity, KU Leuven, Leuven, Belgium

^2^ Brain Imaging and Neural Dynamics Research Group, IRCCS San Camillo Hospital, Venice, Italy

**
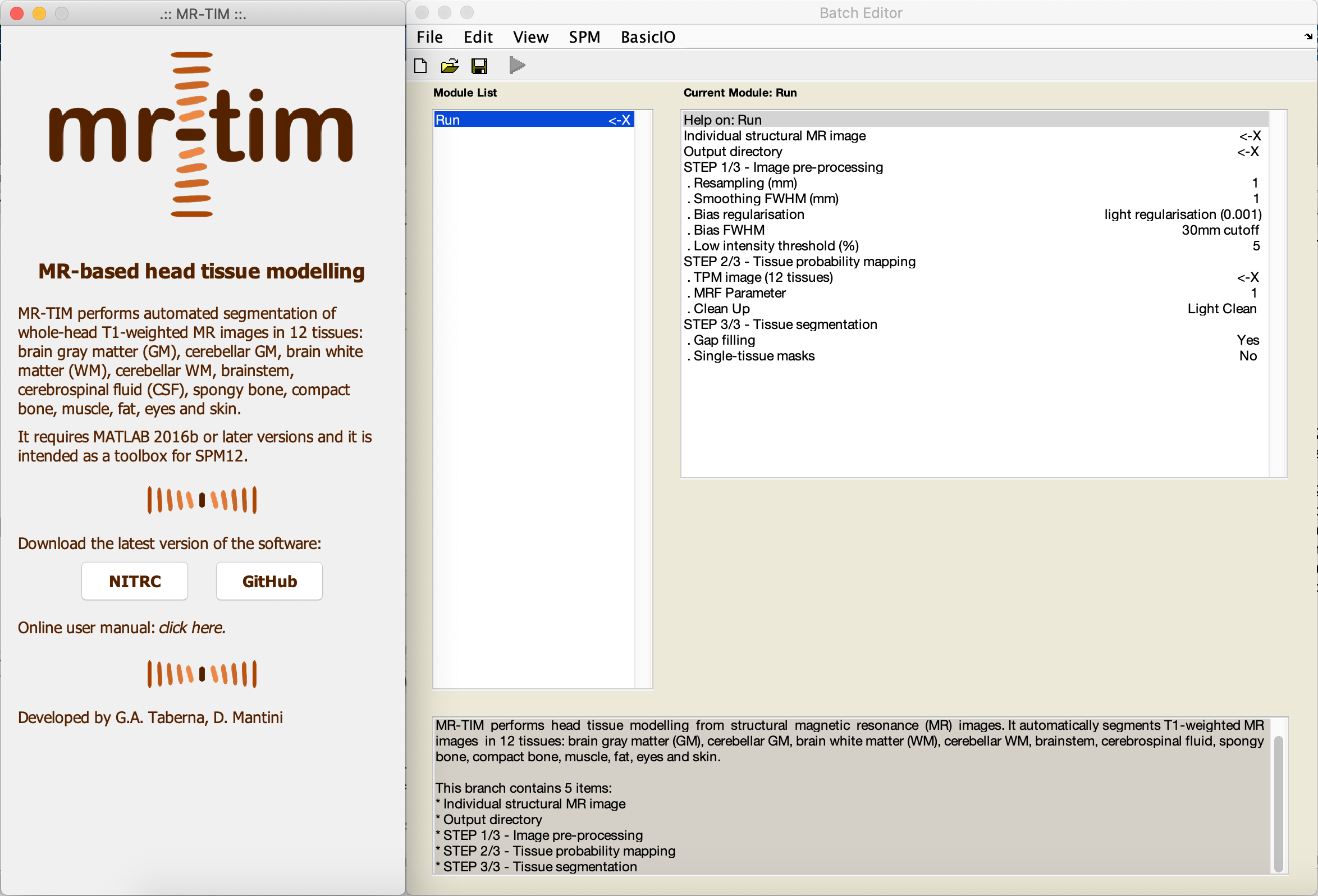
**

**Supplementary Fig. 1**

Screen captures of the MR-TIM toolbox windows. The three main steps of MR-TIM pipeline are: Image pre-processing, Tissue probability mapping and Tissue segmentation


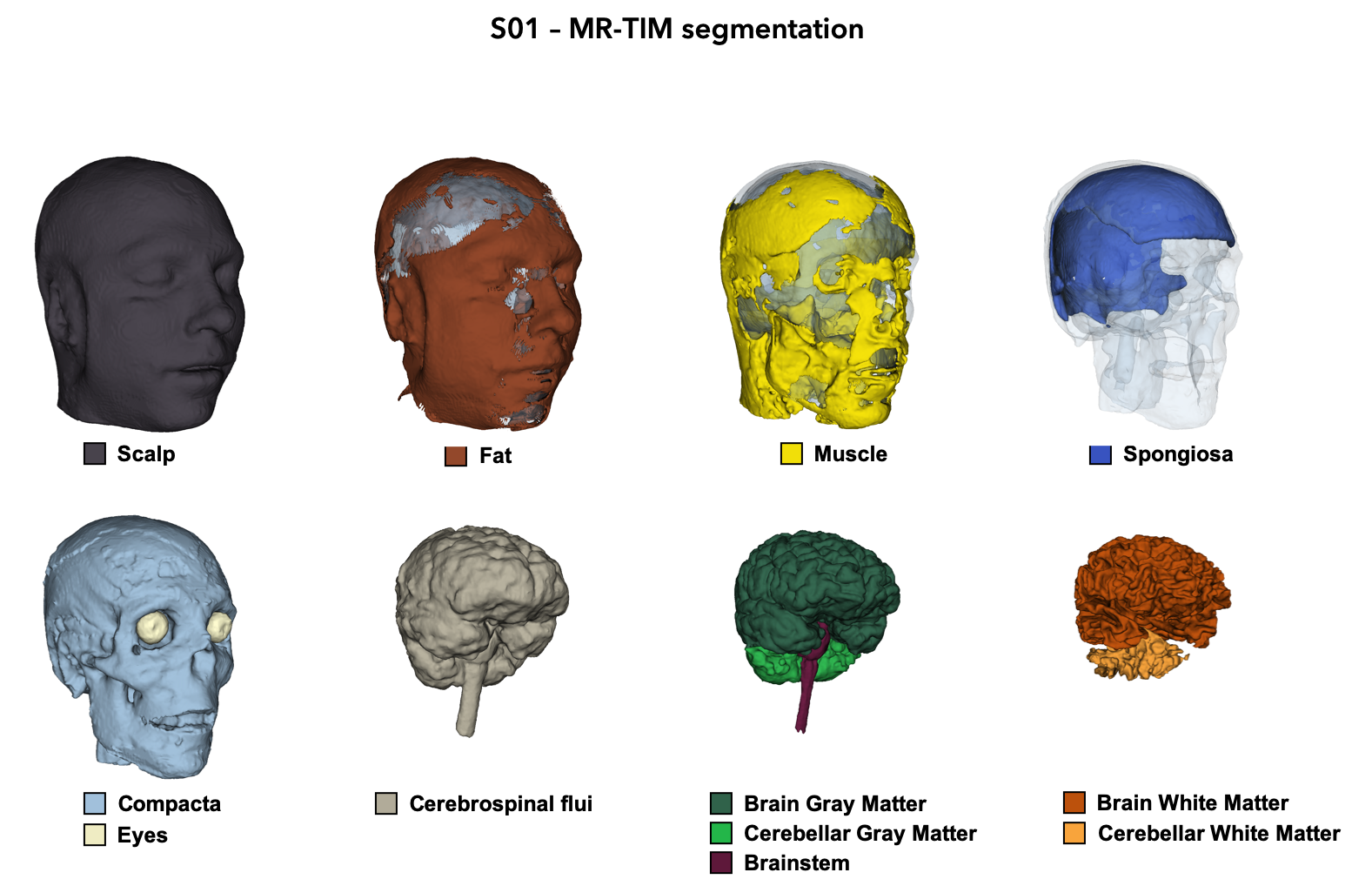


**Supplementary Fig. 2**

Sample output of whole-head automated segmentation produced by MR-TIM. The results obtained on the MR image from subject S01 are shown in 3D space. The 12 tissue classes are represented with different colours


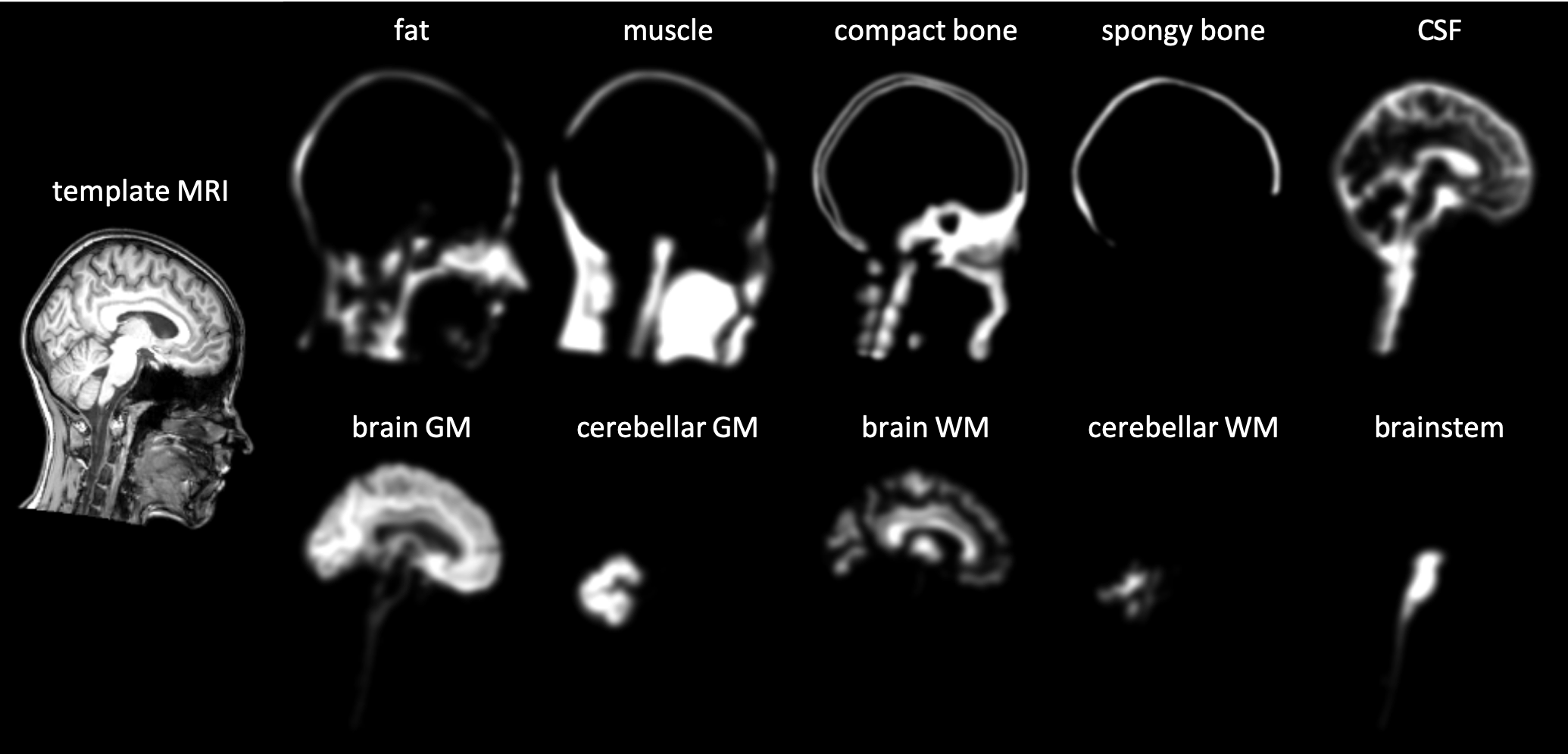


**Supplementary Fig. 3**

Tissue probability maps (TPMs) in the Montreal Neurological Institute (MNI) standard space implemented in MR-TIM. The following ten of the twelve tissue classes are shown in sagittal section (anticlockwise from bottom left): brain grey matter (GM), cerebellar GM, brain white matter (WM), cerebellar WM, brainstem, cerebrospinal fluid, spongy bone, compact bone, muscle, fat


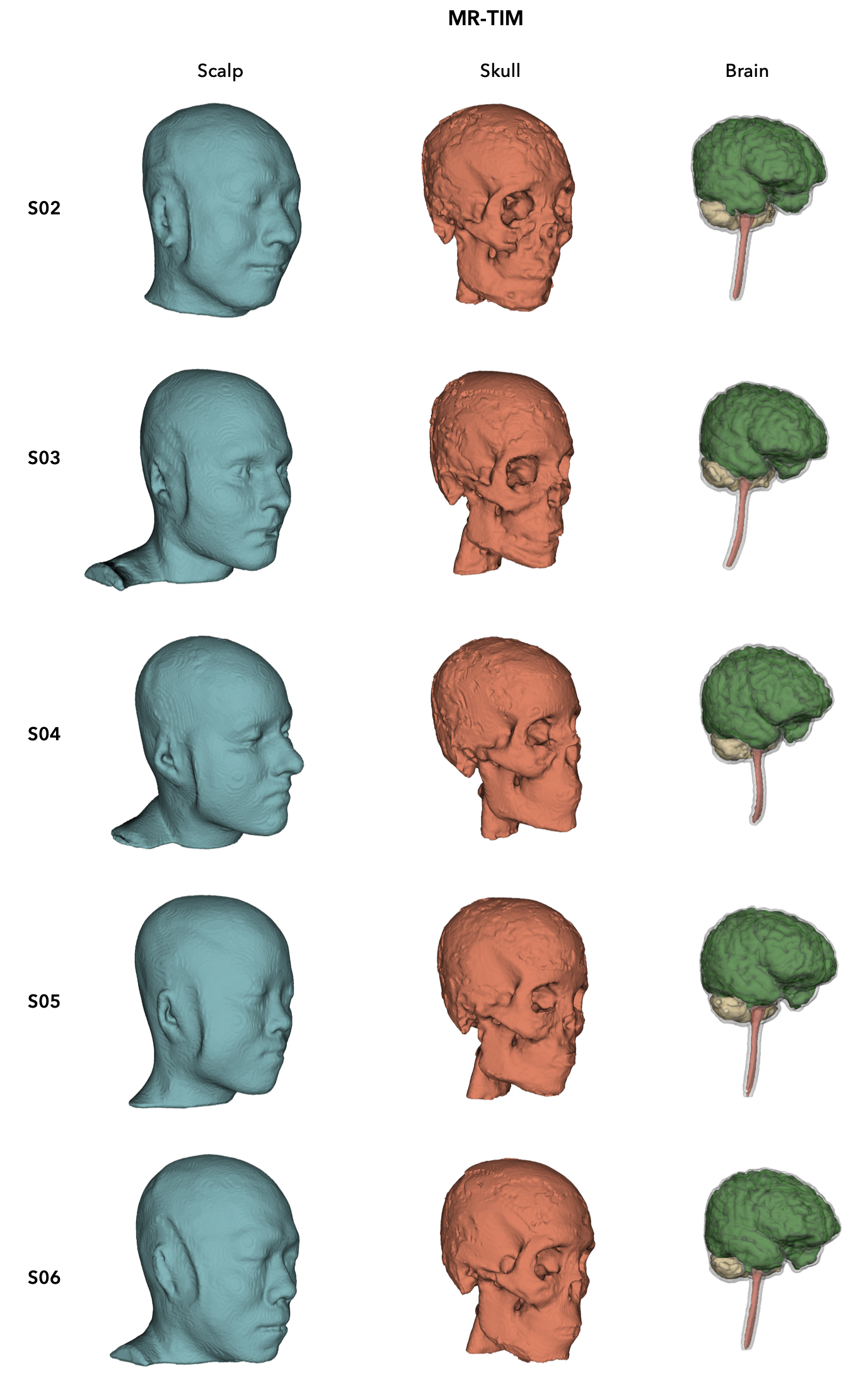


**Supplementary Fig. 4**

3D surfaces of scalp, skull, and brain, estimated by MR-TIM using individual MR images. The results were obtained using the MR image from subjects S02-S06 (from top to bottom)


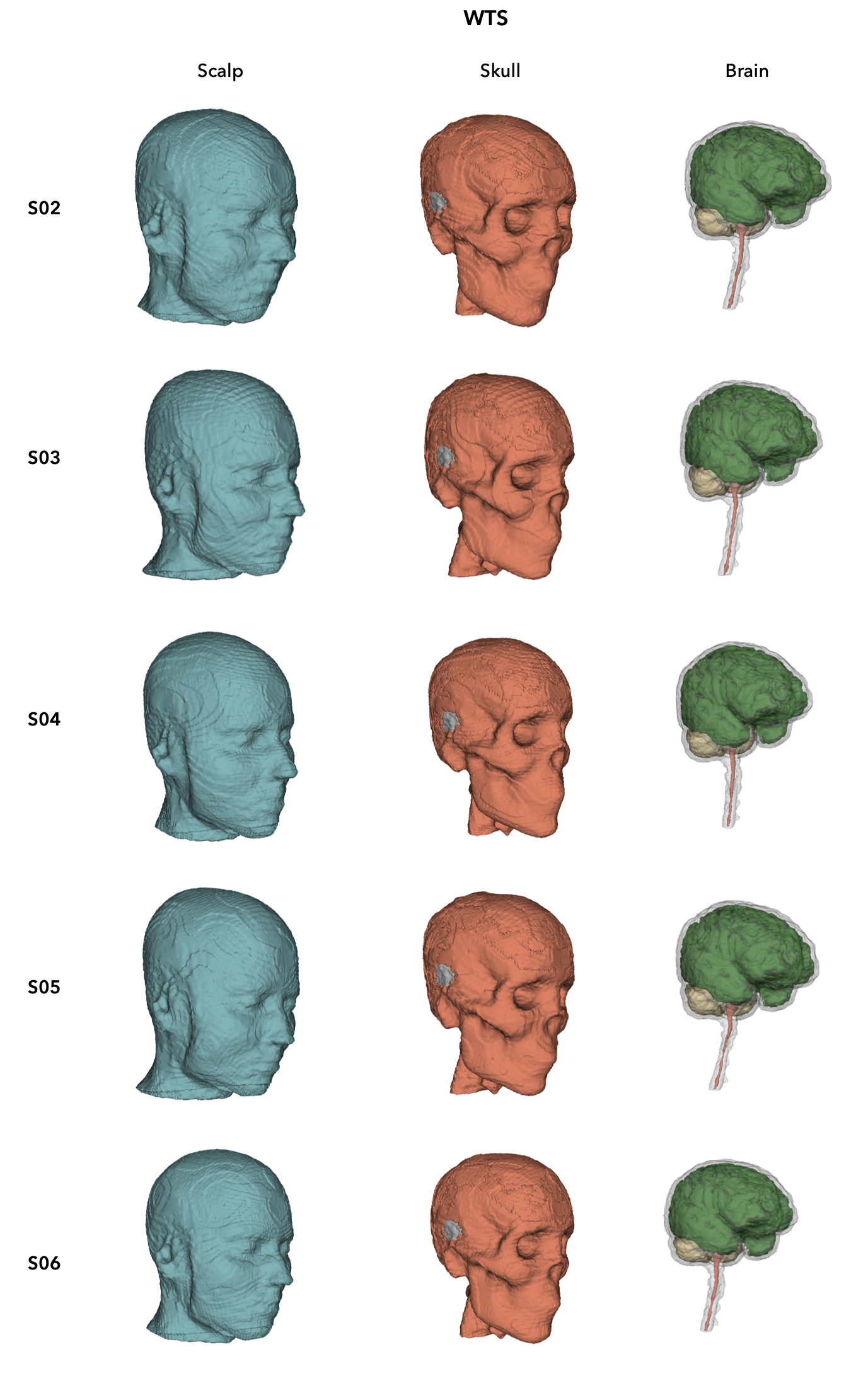


**Supplementary Fig. 5**

3D surfaces of scalp, skull, and brain, estimated by WTS using individual MR images. The results were obtained using the MR image from subjects S02-S06 (from top to bottom)


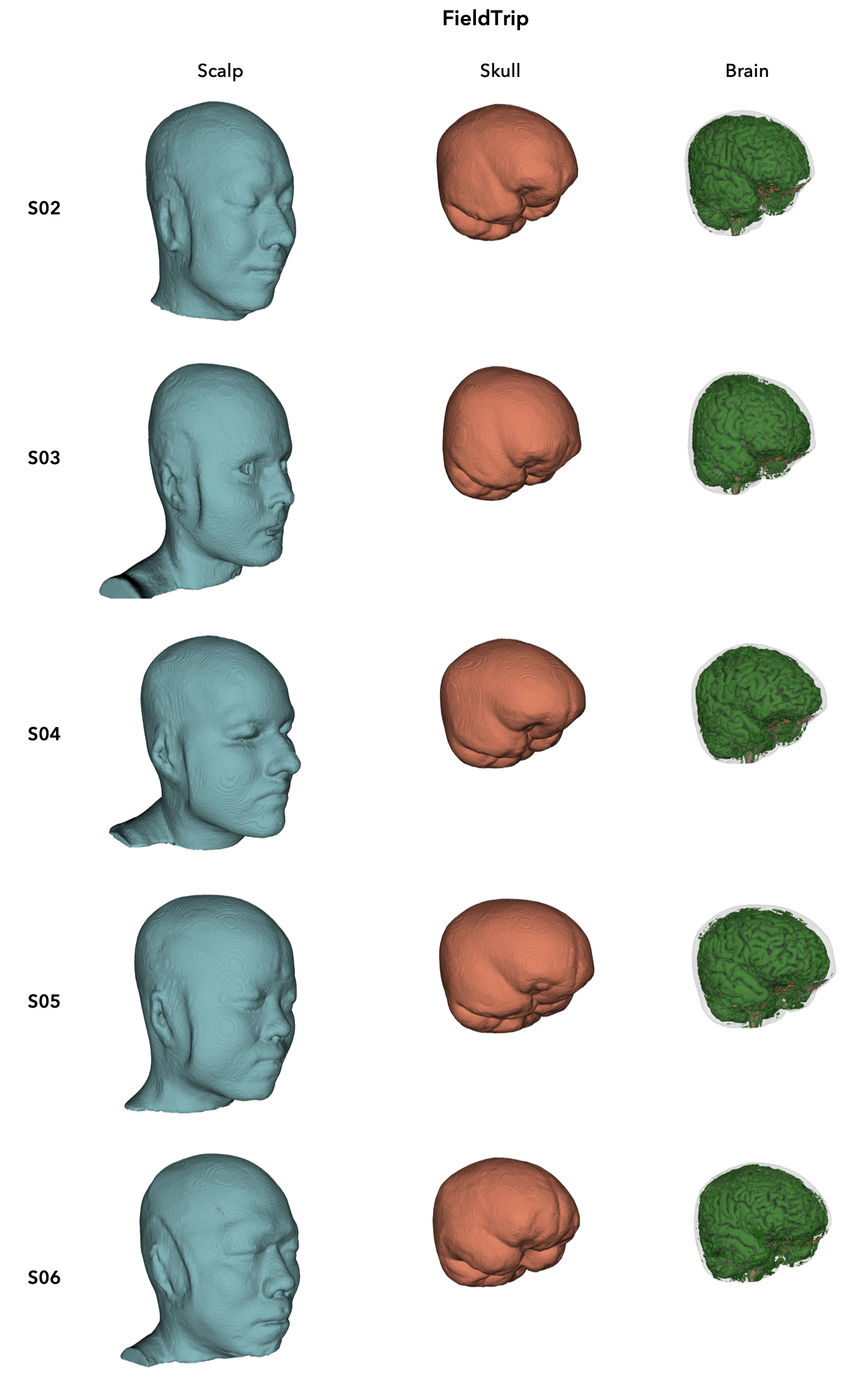


**Supplementary Fig. 6**

3D surfaces of scalp, skull, and brain, estimated by FieldTrip using individual MR images. The results were obtained using the MR image from subjects S02-S06 (from top to bottom)


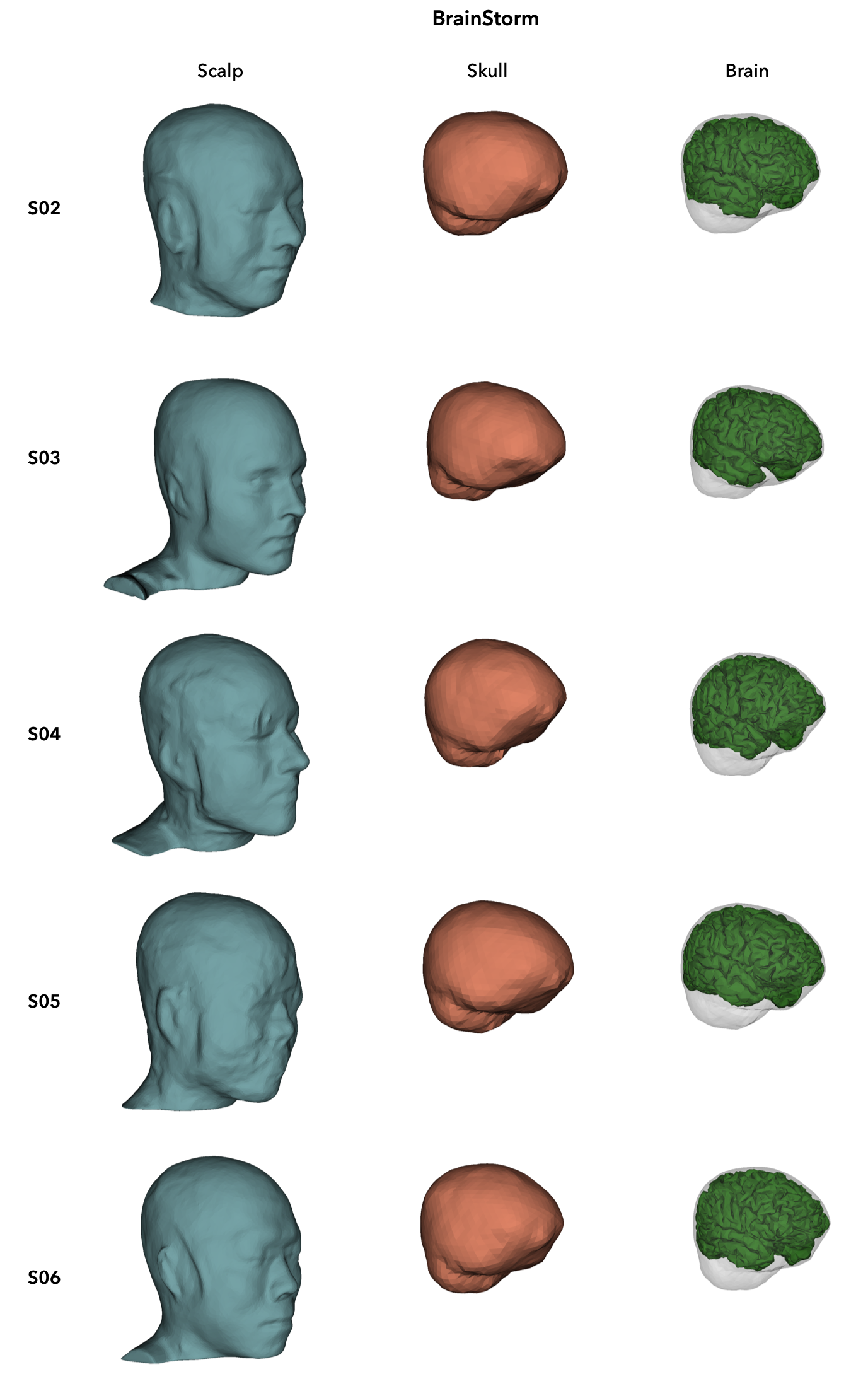


**Supplementary Fig. 7**

3D surfaces of scalp, skull, and brain, estimated by BrainStorm using individual MR images. The results were obtained using the MR image from subjects S02-S06 (from top to bottom)


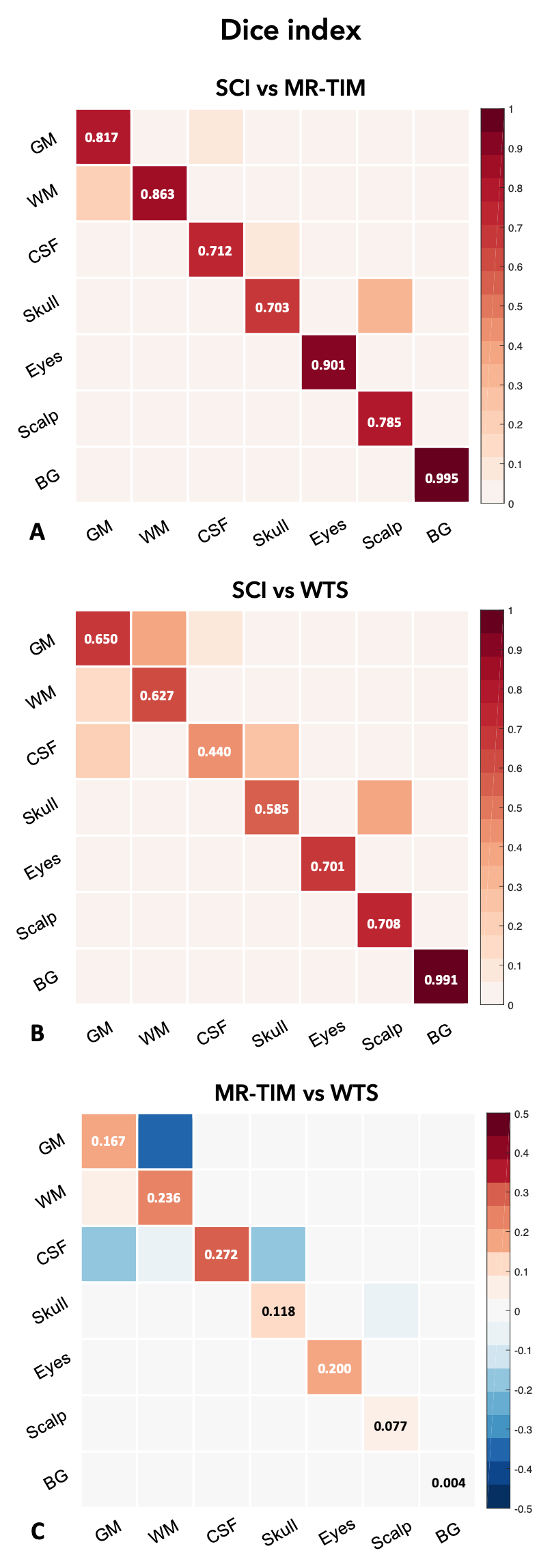


**Supplementary Fig. 8**

Tissue estimation performance of MR-TIM and WTS, measured using the Dice index. The SCI Head Model segmentation is used as reference, and compared against the results produced by (A) MR-TIM and (B) WTS. Each value in the confusion tables represents the Dice index between tissue masks. (C) The difference in the Dice index obtained using MR-TIM and WTS, respectively, is shown as well. GM: grey matter; WM: white matter; CSF: cerebrospinal fluid; BG: background

**
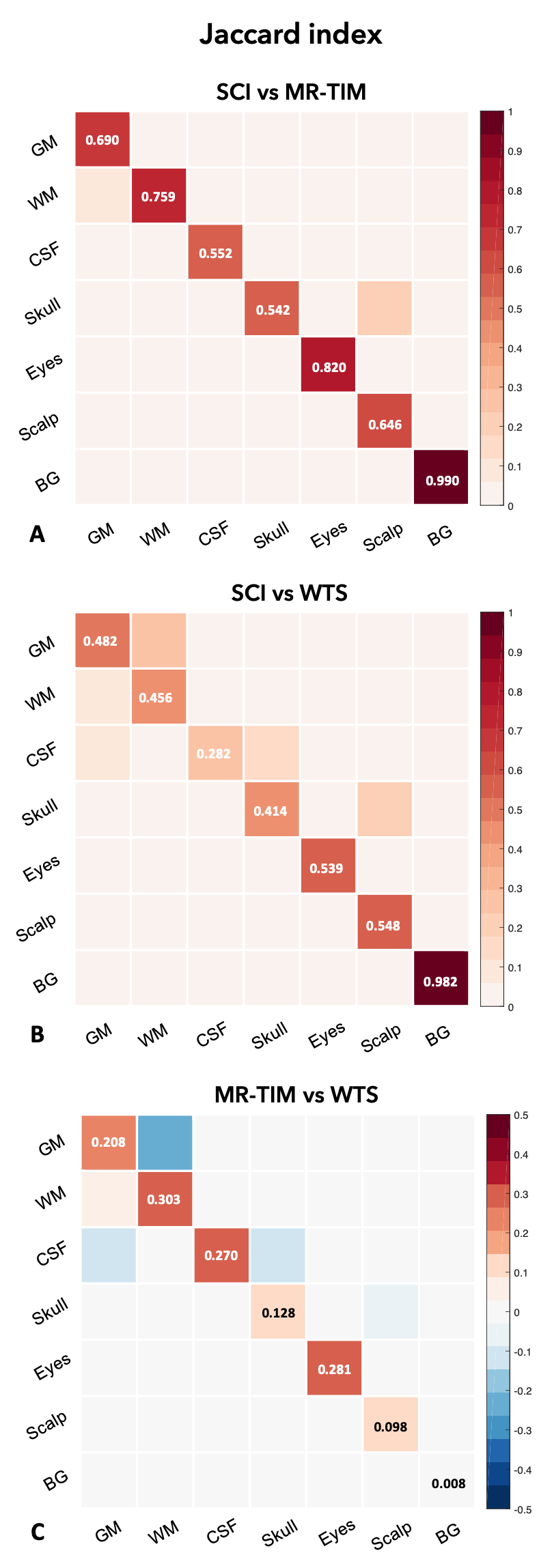
**

**Supplementary Fig. 9**

Tissue estimation performance of MR-TIM and WTS, measured using the Jaccard index. The SCI Head Model segmentation is used as reference, and compared against the results produced by (A) MR-TIM and (B) WTS. Each value in the confusion tables represents the Jaccard index between tissue masks. (C) The difference in the Jaccard index obtained using MR-TIM and WTS, respectively, is shown as well. GM: grey matter; WM: white matter; CSF: cerebrospinal fluid; BG: background

**
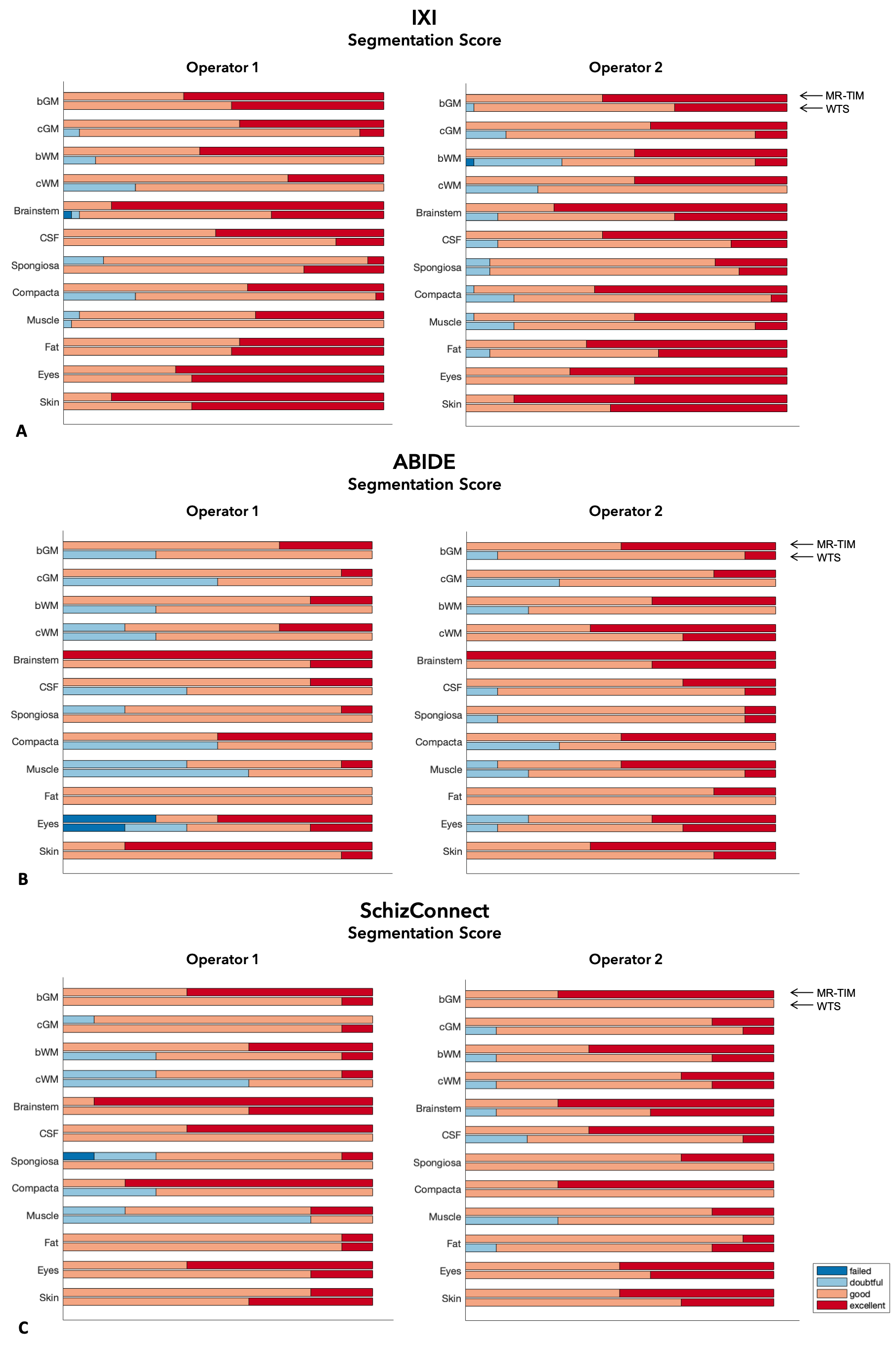
**

**Supplementary Fig. 10**

Qualitative segmentation scores given by the two independent raters, based on the following scale: *excellent* (dark red), *good* (light red), *doubtful* (light blue) and *failed* (dark blue). The stacked bar plots show the distribution of the scores per tissue class. For each of the 12 tissues, the bar plot on the top is related to the score for MR-TIM and the one on the bottom is related to that for WTS. (A) Scores related to the 40 MR images from the IXI dataset; (B) scores related to the 10 MR images from the ABIDE dataset; (C) scores related to the 10 MR images from the SchizConnect dataset
